# Top‐down and bottom‐up characterization of nitrated birch pollen allergen Bet v 1a with CZE hyphenated to an Orbitrap mass spectrometer

**DOI:** 10.1002/elps.201700413

**Published:** 2018-03-14

**Authors:** Sergey Gusenkov, Hanno Stutz

**Affiliations:** ^1^ Department of Biosciences University of Salzburg Salzburg Austria

**Keywords:** Bottom‐up, CZE‐ESI‐Orbitrap MS, Laboratory‐built ESI device, Nitration sites of pollen allergen, Top‐down

## Abstract

Tyrosine (Tyr) residues of the major pollen allergen of birch *Betula verrucosa*, Bet v 1a, were nitrated by peroxynitrite. This modification enhances the allergenicity. Modified tyrosines were identified by analyzing intact allergen variants in combination with top‐down and bottom‐up approaches. Therefore, a laboratory‐built sheath‐liquid assisted ESI interface was applied for hyphenation of CE to an Orbitrap mass spectrometer to localize individual nitration sites. The major focus was on identification of primary nitration sites. The top‐down approach unambiguously identified Tyr 5 as the most prominent modification site. Fragments from the allergen core and the *C*‐terminal part carried up to three potential nitration sites, respectively. Thus, a bottom‐up approach with tryptic digest was used as a complementary strategy which allowed for the unambiguous localization of nitration sites within the respective peptides. Nitration propensity for individual Tyr residues was addressed by comparison of MS signals of nitrated peptides relative to all cognates of homolog primary sequence. Combined data identified surface exposed Tyr 5 and Tyr 66 as major nitration sites followed by less accessible Tyr 158 whereas Tyr 81, 83 and 150 possess a lower nitration tendency and are apparently modified in variants with higher nitration levels.

**Abbreviations**
Bet v 1a: major pollen allergen of birch (*Betula verrucosa*)
BPE: base peak electropherogram
EIE: extracted ion electropherogram
FA: formic acid
HCD: higher energy collision dissociation
ISCID: in‐source collision induced dissociation
NCE: normalized collision energy
NSI: nanospray ionization
pI: isoelectric point
PN: peroxynitrite
SL: sheath liquid
TIC: total ion current
t_m_: migration time
Tyr: tyrosine

## 1 Introduction

Airborne allergies affect more than 20% of the Western European population 1. The pronounced increase in allergic respiratory disorders over the past decade has *inter alia* been related to environmental pollutants (NO_2_ and O_3_) which induce allergen nitration 2, 3. In addition, peroxynitrite (PN) mediated protein nitration takes place during in vivo inflammation 4. Nitration occurs site‐specifically at tyrosine (Tyr) residues, decreases the pK_a_ of the Tyr side‐chain 5 and induces possible conformational changes 6. Nitration propensity depends on surface exposure and molecular environment of the target sites 7. The major allergen of birch (*Betula verrucosa*) pollen, i.e. Bet v 1a, represents a prominent source of respiratory allergies 8. Bet v 1a has a p*I* of 4.95 9, an averaged M_r_ of 17439.58 and contains seven Tyr residues (http://www.uniprot.org/uniprot/P15494) as potential nitration sites. Nitration was shown to increase the allergenicity of Bet v 1a 10. Thus, nitrated allergens represent important tools to reveal underlying mechanisms leading to enhanced allergenicity 10, 11. Due to the absence of commercially nitrated standards, Bet v 1a was nitrated in‐lab with PN which generated closely related nitration variants 12. Beside the nitration grade the localization of nitrated sites might also influence the immunological response to nitrated allergens 10, 11 and has thus to be revealed.

Bottom‐up and top‐down approaches have been applied for the localization of PTMs 13, 14, 15. Peptides derived from bottom‐up analysis possess a higher physicochemical homogeneity than their intact parental proteins which facilitates separation. Particularly in CZE, their adhesion propensity onto the capillary is less pronounced than for proteins 16. On the other hand, digestion is time‐consuming and may induce loss of information: (i) PTM assignment on the peptide level only reveals site occupation averaged over all digested proteins 17. (ii) Additionally, PTMs can also be induced by digestion 18. In top‐down approaches intact proteins are separated up‐stream with subsequent MS fragmentation. This allows for pinpointing PTMs to previously separated intact protein variants and improves quantification 13. For unambiguous PTM localization either peptide derived from fragmentation ideally carries one possible modification site 19. Although gentle top‐down analysis was shown to preserve PTMs better than bottom‐up 20, efficiency of backbone fragmentation depends on the protein sequence 21, structure and the charge state 17. Thus, fragmentation of intact proteins cannot always be achieved to the desired extent 17. Moreover, high mass accuracy enhances the identification credibility of both strategies 13, 15. Although scarcely applied, a few papers showed recently the applicability of CE hyphenated to an Orbitrap mass spectrometer in general and for top‐down approaches as well^22^, ^23^, ^24^, ^25^.

HPLC with UV or MS detection was used for the characterization of nitrated allergens implementing top‐down or bottom‐up strategies 26, 27, 28, 29. Although RP‐HPLC‐ESI‐MS for intact allergens was addressed recently allowing for a distinction of nitrated variants by MS data the related chromatographic separation was not shown 29. Nevertheless, a separation of nitrated and non‐modified proteins by RP‐HPLC was proven elsewhere but did not show the separation of variants with identical nitration level but different modification sites 30. However, a complementary separation method based on charge differences and/or changes in the hydrodynamic radius is favored in the characterization of in‐lab nitrated allergen products. Thereby, CZE hyphenated to MS has demonstrated its remarkable potential over the last decade 31, 32, 33, 34, 35. Among several CE characterizations of allergens carrying PTMs 9, 36, 37, 38, 39, only two CZE methods addressed nitrated proteins/allergens 12, 40, including a CZE‐ESI‐TOF‐MS method 12.

For the discussed characterization strategies mass spectrometers should provide a fragmentation option for top‐down characterization and bottom‐up peptide fragment fingerprinting. Moreover, MS acquisition speed should keep pace with narrow CZE peaks 41. Therefore, CE hyphenation to an Orbitrap mass spectrometer is highly appropriate. Different ESI interfaces have been employed to hyphenate CE to MS, preferably (i) co‐axial sheath liquid (SL)‐ and (ii) sheathless interfaces 35. Alternatively, “liquid junction” or “junction‐on‐the‐tip” 42, 43, 44 have been applied. Recently, a so‐called CESI sprayer was commercially launched based on a prototype of the Moini group 45. Unfortunately, this commercial sprayer is only compatible with a defined CE instrument. The application site for ESI voltage in Orbitrap mass spectrometers prevents the employment of commercial interfaces other than CESI. Dovichi *et al*. described an “electrokinetically pumped SL interface” for coupling CE to Orbitrap mass spectrometers in several applications 41, 46, 47. The SL is EOF‐driven due to the zeta‐potential at the glass emitter resulting in reduced SL flow rates and thus lower dilution 41, 48. However, an external power supply was required and electrospray emitters had to be pulled to assure 2–10 μm id 48.

This work aims to identify preferred nitration sites within Bet v 1a after modification with PN. This will primarily be tackled by a top‐down approach which is innovative and challenging for CE 23 complemented by a bottom‐up strategy. Both approaches employ a front‐end CZE separation hyphenated to an LTQ Orbitrap XL and data will be compared. Since the CESI sprayer is not compatible with our CE systems an interface has to be designed in‐lab based on successful application of “sheath liquid” and “junction‐on‐the‐tip” strategies 43, 48. Thereby, the inherent problem of the voltage configuration in the Orbitrap design has to be surmounted.

## 2 Materials and methods

### 2.1 Reagents

Ammonium bicarbonate (LC‐MS grade), formic acid (FA, 98–100% v/v) and methanol (LC‐MS grade) were all purchased from Sigma‐Aldrich (Vienna, Austria). A 25% v/v ammonia solution (p.A.) and a 1.0 mol/L NaOH solution were purchased from Merck KGaA (Darmstadt, Germany). A 1.0 mol/L HCl solution was obtained from AppliChem (Darmstadt, Germany). Sodium peroxynitrite (PN; >90% in 0.3 mol/L NaOH solution) was purchased from Cayman Chemical (Ann Arbor, MI, USA). Ultrapure water with a resistivity >18.2 MΩ.cm was supplied by a Milli‐Q Plus 185 system (Millipore S.A., Molsheim, France). MS calibration mixture (Pierce™ LTQ ESI Positive Ion Calibration Solution) for the positive mode was purchased from Thermo Fisher Scientific (San Jose, CA, USA). Trypsin (Sequencing Grade Modified) which was delivered with a resuspension buffer and Glu‐C (Sequencing Grade) were purchased from Promega (Madison, WI, USA).

### 2.2 Sample preparation and digestion

#### 2.2.1 Sample nitration

Recombinant Bet v 1a was produced in‐house 49, provided as a 1.6 mg/mL Bet v 1a solution in 20 mmol/L ammonium bicarbonate pH 7.4 and stored at −20°C. Prior to allergen nitration a buffer exchange to 10 mmol/L ammonium bicarbonate (adjusted to pH 7.50 with FA) was done by ultrafiltration via an Amicon centrifugal filter unit with a molecular cut‐off of 10 kDa (Merck Millipore, Cork, Ireland). Nitration was accomplished by addition of PN to obtain a 1:1 molar ratio between the nitration reagent and the Tyr residues in Bet v 1a. This ratio primarily generates single nitrated Bet v 1a variants 12 and is thus favorable for the elucidation of primary nitration sites. After stirring for 1 h at room temperature, the reaction was stopped by centrifuging the mixture with 14 000 × *g* through a 4 mL Amicon centrifugal filter with a 10 kDa cut‐off to separate PN from the allergen. Retained nitrated allergens were washed three times with 500 μL 10 mmol/L ammonium bicarbonate (pH 7.50) and subsequent centrifugation with 14 000 × *g*, respectively. A final volume of 500 μL was adjusted with 10.0 mmol/L ammonium bicarbonate (pH 7.50).

#### 2.2.2 Digestion of nitrated allergen for bottom‐up analysis

The sample was incubated at 60°C for 1 h. As Bet v 1a contains no cysteines and thus no disulfide bridges, reduction and alkylation steps were not implemented in the digestion protocol. Nitrated Bet v 1a (1.6 mg/mL in 10 mmol/L ammonium bicarbonate) was mixed with trypsin protease in a 1:50 w/w enzyme to substrate ratio. After the thermal denaturation the first half of the required trypsin portion was added and the sample was incubated at 37°C for 2 h. Afterwards, the second trypsin portion was added and the protein was incubated at 37°C for another 4 h. Finally, the digested sample was dried in a vacuum centrifuge (20.0 mbar at 45°C with 2000 rpm) and stored at ‐20°C. Prior to analysis the dried sample was reconstituted in 10.0 mmol/L ammonium bicarbonate, pH 7.50.

### 2.3 Instrumentation

#### 2.3.1 Capillary electrophoresis

CZE separations were performed with a P/ACE™ System MDQ CE of Beckman Coulter (Brea, CA, USA) applying a bare fused‐silica capillary of 50 μm id and 375 μm od from Polymicro Technologies (Phoenix, AZ, USA) with a total length (L_T_) of 106.3 cm. Prior to their first use, capillaries were conditioned with 1.0 mol/L NaOH (10.0 min), ultrapure water (15.0 min), 0.10 mol/L HCl (10.0 min), and BGE (20.0 min), all with 1500 mbar 50. Every three runs the capillary was rinsed as described elsewhere 12. For capillary conditioning and rinsing the nanospray ionization (NSI) source was decoupled from the mass spectrometer. The sample was injected with 35 mbar for 10.0 s. CZE separations were performed at +20.0 kV with a ramp time of 1.0 min and the cathode situated at the capillary outlet. Separation temperature was set to 25.0°C. Capillary was stored in BGE at room temperature overnight.

#### 2.3.2 In‐lab designed ESI interface

**Figure 1:**
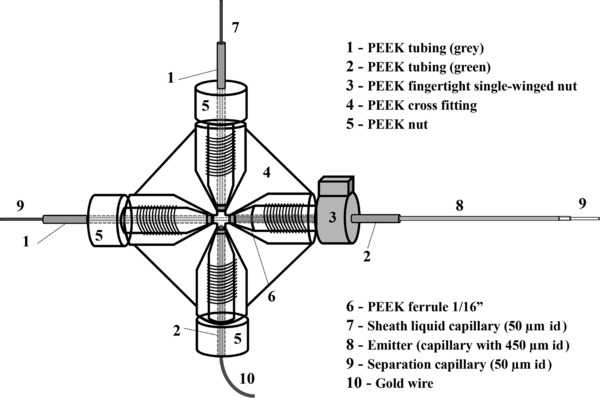
Scheme of the in‐lab designed ESI interface applicable for hyphenation of CE to an Orbitrap mass spectrometer.

The CE hyphenation to the Orbitrap mass spectrometer was established by means of an in‐lab designed SL interface (Fig. 1). This cost‐efficient SL‐sprayer provides general compatibility with Orbitrap mass spectrometers but in principle also with other commercial MS systems. Contrary to the Dovichi design 48, neither the application of an additional external power supply nor the preparation of a tapered emitter is required. Further details of the sprayer are given in Supporting Information 1. The interface was integrated into the mounting device of the commercial NSI probe from Thermo Fisher Scientific via a single‐winged nut.

#### 2.3.3 LTQ Orbitrap XL

Calibration of the LTQ Orbitrap XL was done with the ESI probe by introducing the Pierce™ LTQ ESI Positive Ion Calibration Solution in the positive ionization mode at 10.0 μL/min via the syringe pump of the MS. Tuning was done with the laboratory‐built SL interface applying a 0.16 mg/mL nBet v 1a solution by the CE system via a 50 μm id fused‐silica capillary at 100 mbar. Simultaneously, SL (Section 2.3.4) was introduced with 3.0 μL/min and a spray voltage of +2.5 kV was applied. The same tune file was used for top‐down and bottom‐up experiments. The following MS settings were selected, respectively: tube lens was 120.0 V, capillary voltage and temperature were 43.0 V and 200°C, respectively, and the automatic gain control target was set to 1 × 10^6^. The maximum injection time was 50 ms and one microscan was recorded.

Top‐down experiments were performed on single nitrated variants by isolating *m/z* 1030.4 (referring to z = +17) with an isolation width of *m/z* 4.0. This resulted in a clear isolation of a desired m/z 1029.5 and its isotopic pattern. Fragmentation was done in higher energy collision dissociation (HCD) cell with a normalized collision energy (NCE) of 20.0 and subsequent analysis of fragments in the Orbitrap mass analyzer. Scan range was *m/z* 200–2000. In‐source collision induced dissociation (ISCID) of 15.0 V was applied to improve the quality of mass spectra by removing adducts.

Bottom‐up experiments were performed with data dependent acquisition implementing two scan events. Thereby, a full scan with *m/z* 400–2000 and 30 000 resolving power at *m/z* 400 was succeeded by data dependent MS/MS of the respective target peptide ions or (if not detected) the most intense ions with a scan range of *m/z* 100–2000 applying HCD with a NCE of 35.0.

#### 2.3.4 CZE‐ESI‐Orbitrap‐MS(/MS) analysis

Prior to the ramping of the CZE separation voltage, an ESI voltage of +2.5 kV was applied to promote spraying of the introduced SL. Parallel to the ramping of the CZE separation voltage to 20.0 kV, the ESI voltage had to be reduced gradually to 0.80 ± 0.20 kV to maintain a stable spray. For all CZE‐ESI‐Orbitrap XL MS(/MS) measurements 10.0 mmol/L ammonium bicarbonate with pH 7.50 (BGE) and a SL containing 75.0% methanol, 24.9% ultrapure water and 0.1% FA (all v/v/v) 12 were used.

#### 2.3.5 Data analysis

Data for top‐down experiments were treated by Xcalibur 2.2 SP 1.48. High resolution mass spectra were deconvoluted with Xtract algorithm with a resolving power of 60 000 at 400 m/z and with a signal‐to‐noise threshold of 2 with AvaragineNoSulfur software option. Extracted ion electropherograms (EIEs) were smoothed with Boxcar option 7 and masses were extracted with a 10 ppm tolerance. The sequence coverage map for top‐down fragmentation experiments was generated with the ProSightPTM online tool (https://prosightptm2.northwestern.edu/) provided by the Kelleher Research Group (Northwestern University, Evanston, IL, USA) 51. PeptideMass program (http://web.expasy.org/peptide_mass/) from Expasy (SIB Bioinformatics Resource Portal) 52, 53 was used for predicting the tryptic peptide masses of Bet v 1a and corresponding nitrated variants excluding missed cleavages. The amino acid sequence of Bet v 1a was from the UniProt data base (http://www.uniprot.org/uniprot/P15494).

## 3 Results and discussion

### 3.1 Performance of in‐lab designed ESI sprayer

**Figure 2:**
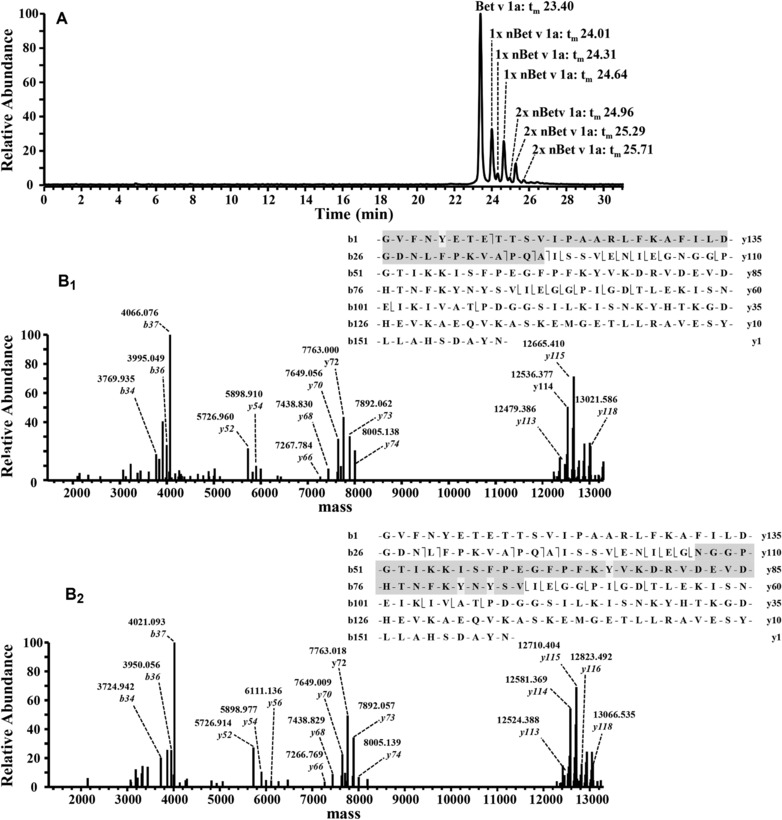
Top‐down characterization of prominent single nitrated Bet v 1a species. Nitration was done with PN in a 1:1 molar ratio between the nitration reagent and the Tyr residues. (A) BPE of nitrated Bet v 1a indicating single (1x) and double (2x) nitrated Bet v 1a variants. The major peak at 23.40 min refers to non‐modified recombinant Bet v 1a. CZE: 10 mmol/L ammonium bicarbonate, pH 7.50. Separation: 20.0 kV, 25.0°C. (B) Deconvoluted HCD‐MS/MS spectra. Inserts depict corresponding fragment ions with gray section annotating fragments carrying the nitrated tyrosine residue, respectively. (B_1_) Fragment ions for single nitrated species at 24.01 min. (B_2_) Fragment ions for single nitrated species at 24.64 min. Due to a deficiency of fragmentation by HCD within the protein core between Asp 47 and Val 85 (marked in grey in the insert) *y*‐ions between *y74* and *y113* are missing. Thus, possible nitration sites for this selected single nitrated variant comprise Tyr 66, 81 or 83.

A representative base peak electropherogram (BPE) for nitrated Bet v 1a is given in Fig. 2A. Compared to the commercial Agilent coaxial interface 12, the in‐lab developed ESI interface provided an equivalent profile of nitrated Bet v 1a variants. The performance of the in‐lab designed sprayer was evaluated by five consecutive injections of the tryptic digest of nitrated Bet v 1a. CVs of migration times were 0.09–0.57% (*n* = 5). CVs for peak areas and peak heights were <13.5% and < 7.3%, respectively. Higher CVs were only encountered for peaks with partial resolution or low abundance. Details are given in Supporting Information 2.

### 3.2 Top‐down experiments

The achieved CZE separation of isobaric nitration variants of Bet v 1a is related to their different p*I* values. Depending on their position within the primary sequence nitrated Tyr (nTyr) residues apparently take slightly different pK_a_. Alternatively, minor changes in the hydrodynamic radius might be induced by nitration as well. Both effects explain the observed differences in the effective electrophoretic mobility of isobaric species. Individual nitration sites are addressed by a top‐down approach. The primary focus is on sites with high nitration propensity. Three single nitrated species (1x nBet v 1a) were electrophoretically resolved and constitute ideal candidates for a top‐down localization of primary nitrated sites. Initial nitration events apparently occur in parallel at Tyr residues with high nitration propensity. Two 1x nBet v 1a are present in high abundance, whereas one constitutes a minor peak (Fig. 2A). A combinatory nitration of high propensity Tyr residues is assumed to provide the observed two‐fold nitrated variants (2x nBet v 1a) (Fig. 2A). Modifications of additional Tyr residues with lower nitration tendency will hierarchically contribute to low abundant variants of higher nitration grades.

ISCID, collision induced dissociation (CID), and HCD were tested for fragmentation in top‐down experiments. Compared to CID, ISCID did not provide an appropriate number of fragments (data not shown) which also corresponds to literature 17, 54. However, low energy ISCID was co‐applied in all top‐down experiments to improve the quality of spectra. CID fragmentation provides approximately an equal number of b‐ and complementary y‐ions in contrast to HCD which preferentially generates y‐ions 55 as experimentally confirmed (Fig. 2B_1,2_). A NCE of 20 was selected as an optimum for HCD fragmentation of intact nBet v 1a variants. Compared to CID this resulted in a higher number of fragments derived from the protein core of Bet v 1a which could contain important diagnostic fragments. All b‐ions given in Fig. 2B_1,2_ are diagnostic as they only cover Tyr 5.

Top‐down results were evaluated with ProSight PTM which compares experimental with theoretical MS/MS spectra, assuming nitration of Tyr for 1x nBet v 1a variants. Figure 2 depicts the BPE of the nitrated sample and MS/MS spectra for the two most prominent 1x nBet v 1a variants. Nitration at Tyr 5 was confirmed for 1x nBet at 24.01 min (Fig. 2A) by top‐down analysis (Fig. 2B_1_) based on diagnostic b‐ions *b*34, *b*36 and *b*37. Mass increments of +44.99 Da are related to nitration (Fig. 2B_2_). For the second prominent 1x nBet v 1a peak at 24.64 min (Fig. 2A) top‐down data indicate one nitration between Asn 47 and Val 85 which is apparent from mass shifts of +44.99 Da for *y*113‐*y*118, but not for *y*52‐*y*74 (Fig. 2B_1,2_). This sequence contains three possible nitration sites, i.e. Tyr 66, 81 or 83. Neither CID nor HCD demonstrated a diagnostic fragment between Asn 47 and Val 85 (Fig. 2B_2_) for this nitrated species which prevents the unambiguous assignment of the nitrated site. The selection of other charge states for a fragmentation of this nitrated variant was neither successful (data not shown). A lack of fragmentation in the protein core was also described elsewhere 17. A corresponding problem was observed for 1x nBet v 1a at 24.31 min (Fig. 2A). However, this time the nitrated fragment is situated in the *C*‐terminal protein part and carries three Tyr residues. According to the y‐ions the nitration might have occurred at Tyr 120, 150 or 158 (data not shown).

Top‐down experiments revealed Tyr 5 as predominant nitration site in single nitrated species (Fig. 2A, peak at 24.01 min). As postulated previously, the most prominent 2x nBet v 1a variant (Fig. 2A, peak at 25.29 min) most likely combines nitration at Tyr 5 and the second high‐propensity site, i.e. Tyr 66, 81, or 83. However, top‐down experiments for the prominent double nitrated variant resulted in b‐and y‐ions similar to single nitrated variants with equal deficiency in nitration assignment to Tyr 66, 81, or 83 (data not shown). Application of electron transfer dissociation as an alternative fragmentation technique, alone or in combination with HCD, could possibly provide an improved sequence coverage and site identification 56, as it cleaves along the backbone in a size‐ and sequence‐independent manner 57. However, this fragmentation option was not available on our instrument.

### 3.3 Bottom‐up experiments

Since some top‐down fragments contained more than one Tyr, an unambiguous assignment of high propensity nitration sites was not possible in all instances. Thus, a complementary bottom‐up approach was applied. Based on an in‐silico simulation with the “PeptideMass” program, trypsin and Glu‐C provided best sequence coverage and with the exception of two Tyr pairs covered all Tyr residues on individual peptides. Experimental CZE‐ESI‐Orbitrap XL MS data revealed a low digestion efficiency of pricy Glu‐C enzyme resulting in a mixture of non‐digested intact proteins and peptides (data not shown). Hence, trypsin was the most expedient enzyme for further bottom‐up experiments.

**Figure 3:**
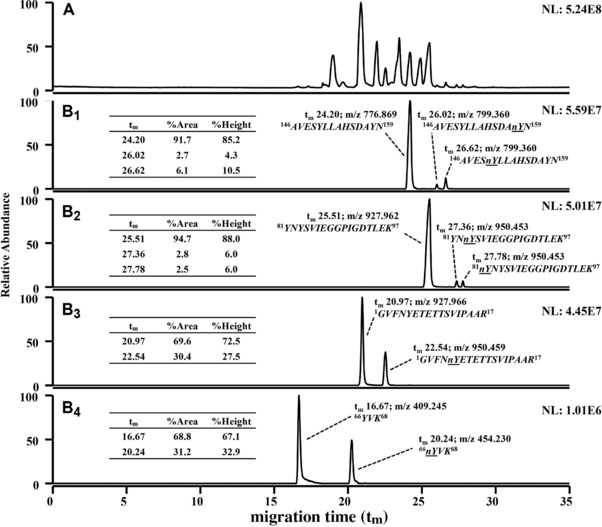
Bottom‐up characterization of nitrated Bet v 1a after tryptic digest. The nitrated sample corresponds to Fig. 2. (A) Total ion current electropherogram of tryptic digest. (B_1–4_) represent EIEs of Tyr containing tryptic peptides. Peaks refer to non‐modified tryptic peptides and their nitrated homologs, respectively. Peptides carrying a nitration show a mass increment of +44.99 Da. *^66^YVK^68^* is single charged, whereas all other depicted peptides carry two charges. Thus, the observed mass yield by nitration is only half in the latter cases. Nitration propensity was evaluated by relative areas and heights derived from the respective ratio between the target peptide and the sum of all cognate peptides as indicated in the tabular inserts. nY refers to nitrated Tyr.

In order to understand the profile of intact 1x nBet v 1a variants and their relative intensities (Fig. 2A) an evaluation of the abundance of nitrated tryptic peptides is supportive (Fig. 3). All nitrated peptides possessed higher migration times (t_m_) than their non‐modified cognates (Fig. 3B_1–4_) due to the pK_a_ change of nitrated Tyr. This serves as a co‐decisive criterion for nitration. The tryptic digest generates two non‐modified isobaric peptides, i.e. *^1^GVFNYETETTSVIPAAR^17^* and *^81^YNYSVIEGGPIGDTLEK^97^*, with three derived isobaric nitrated peptides (Fig. 3B_2,3_). However, their different pIs allow for a CZE separation. This alliance of a charge‐related front‐end separation of high‐efficiency (CZE) with high mass accuracy and resolution of an Orbitrap mass spectrometer proves its virtue for distinguishing closely related peptides and elucidating site‐specific nitration.

Seven potential nitration sites were situated on five tryptic peptides. Tyr 81 and 83, but also Tyr 150 and 158 (Fig. 3B_1,2_) were pairwise located on a tryptic peptide, respectively. Tyr 5, 66 (Fig. 3B_3,4_) and 120 (not shown) were localized on individual peptides. Their nitration can be addressed unambiguously by MS/MS (Section 3.3.1). Naturally, primary sequences and lengths of all five Tyr‐carrying tryptic peptides differ. Thus, different ionization efficiencies with concomitantly divergent signal heights and areas in MS cannot be excluded for these peptides. Consistently, a relative quantification by comparing absolute abundances of nitrated peptides would be misleading. Instead, signals of nitrated peptides have to be related to their non‐modified homologs, respectively. Even in case of different ionization efficiencies of nitrated peptides and their non‐nitrated siblings the relative response was shown to be maintained 58. Comparisons of signal heights and areas for nitrated and non‐nitrated peptide siblings allowed to estimate the relative nitration propensity of either site (Fig. 3B_1–4_). In total, six Tyr residues (namely Tyr 5, 66, 81, 83, 150, 158) out of seven were found to be nitrated (Fig. 3) as confirmed by MS/MS (see Section 3.3.1). Experimental data did not reveal nitration of Tyr 120. Therefore, the corresponding peptide was not shown.

#### 3.3.1 Peptide fragmentation for nitration assignment

**Figure 4:**
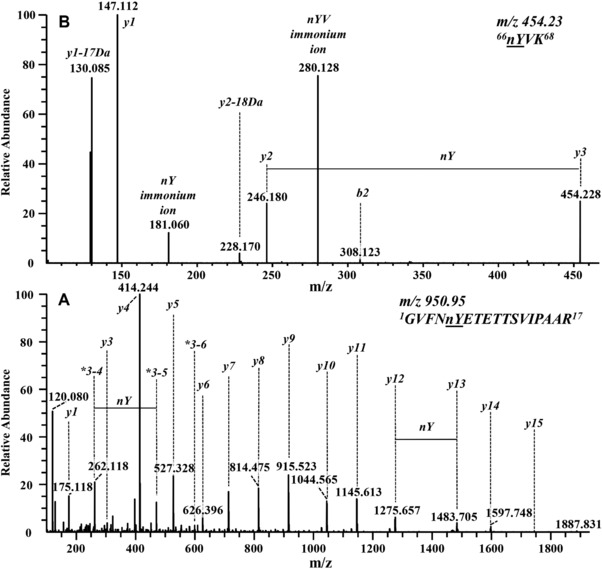
HCD‐MS/MS spectra of nitrated variants of peptides (A) *^1^GVFNYETETTSVIPAAR^17^* (see Fig. 3B_3_) and (B) *^66^YVK^68^* (see Fig. 3B_4_) derived from a tryptic digest of nitrated Bet v 1a. The depicted series of *b‐* and *y‐*ions reveal nitration of (A) Tyr 5 for *^1^GVFNYETETTSVIPAAR^17^* and (B) Tyr 66 for *^66^YVK^68^*. The fragment with m/z 181.060 represents the 3‐nitrotyrosine immonium ion. * annotate internal fragments (number indicates the position of amino acids).

**Figure 5:**
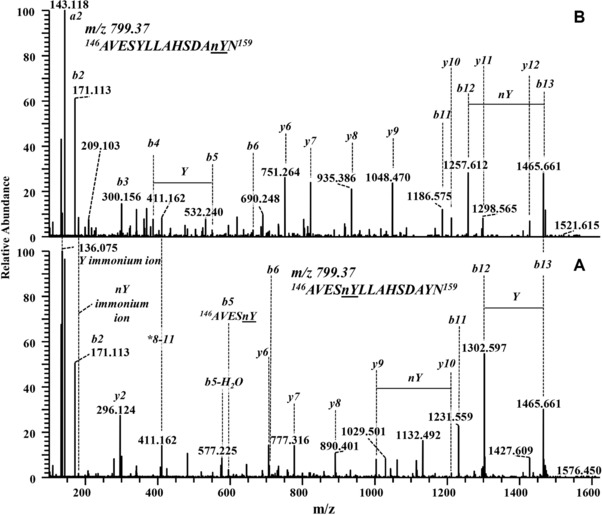
HCD‐MS/MS spectra of two nitrated variants of the peptide *^146^AVESYLLAHSDAYN^159^* (see Fig. 3B_1_) derived from a tryptic digest of nitrated Bet v 1a. Depicted series of *b‐* and *y‐*ions indicate nitration of (A) Tyr 150 and (B) Tyr 158 on peptide *^146^AVESYLLAHSDAYN^159^*. The fragment with m/z 136.075 represents the tyrosine immonium ion.

**Figure 6:**
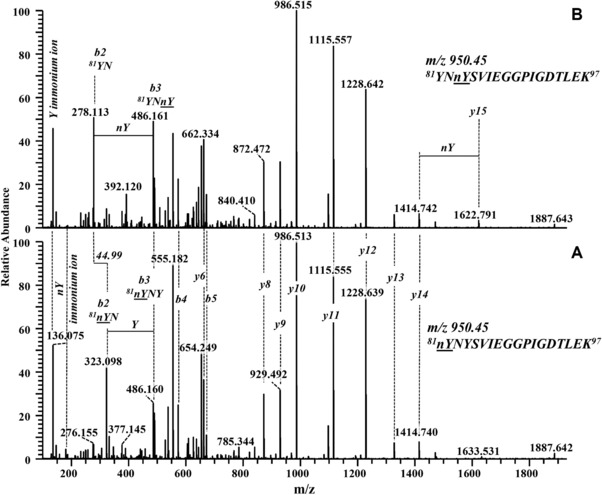
HCD**‐**MS/MS spectra of two nitrated variants of the peptide *^81^YNYSVIEGGPIGDTLEK^97^* (see Fig. 3B_2_) derived from a tryptic digest of nitrated Bet v 1a. Depicted series of *b‐* and *y‐*ions indicate nitration of (A) Tyr 81 and (B) Tyr 83 on peptide *^81^YNYSVIEGGPIGDTLEK^97^*. The fragment with m/z 136.075 represents the tyrosine immonium ion.

The HCD‐MS/MS spectrum of the data‐dependent acquisition of the peptide *^1^GVFNnYETETTSVIPAAR^17^* with nitrated Tyr 5 (Fig. 3B_3_) predominantly consisted of y‐ions (Fig. 4A). The fragmentation spectrum also contained internal fragments 59, annotated as 3–4, 3–5 and 3–6 (Fig. 4A), and b‐ions of very low intensity (not annotated). Nitration of Tyr 5 is confirmed from *y12, y13* and internal fragments 3–4, 3–5. The MS/MS spectrum for *^66^YVK^68^* (Fig. 3B_4_) shows a nitration at Tyr 66 as proven by y‐ions *y2, y3*. The presence of a 3‐nitrotyrosin (nY) immonium ion at m/z 181.060 (Fig. 4B) provides an additional proof for nitration 60. Two of the tryptic target peptides (Fig. 3B_1,2_) contained two Tyr residues, respectively, i.e. *^81^YNYSVIEGGPIGDTLEK^97^* including Tyr 81 and 83 and *^146^AVESYLLAHSDAYN^159^* with Tyr 150 and 158. MS/MS spectra of both nitrated variants of *^146^AVESYLLAHSDAYN^159^* with single nitration in different positions are compared (Fig. 5A and B). Both spectra contain immonium ions of Tyr and of nitrated Tyr. The MS/MS spectrum referring to the peptide with t_m_ 26.02 (Fig. 3B_1_) proves a nitration at Tyr 158 as confirmed by the *b12, b13* ion series (Fig. 5B). The MS/MS spectrum of the tryptic peptide with t_m_ 26.62 min (Fig. 3B_1_) indicates nitration of Tyr 150 as verified by *b5* and *y9, y10* (Fig. 5A). A comparison of relative peak areas of all nitrated peptides (Fig. 3B_1_‐B_4_) indicated Tyr 150 as the third‐prominent nitration site. The nitration propensity of Tyr 81 and 83 within the sequence *^81^YNYSVIEGGPIGDTLEK^97^* was addressed by a corresponding bottom‐up approach. Immonium ions of Tyr and nitrated Tyr were both present but the former dominates. MS/MS spectra for the tryptic peptide at 27.78 min (Fig. 3B_2_) prove nitration of Tyr 81 as confirmed by *b2, b3*‐ions (Fig. 6A). MS/MS spectra for the tryptic peptide at 27.36 min (Fig. 3B_2_) confirmed nitration of Tyr 83 by *y14, y15* and *b2, b3*‐ions (Fig. 6B). The peak area ratios between the nitrated peptides, respectively, and the sum of all cognate peptides (Fig. 3B_2_) demonstrate a rather low nitration occurrence of Tyr 81 and 83. This is apparently due to their low accessibility since both are localized in a hydrophobic cavity 61.

### 3.4 Comparison of top‐down and bottom‐up results

Top‐down analysis revealed Tyr 5 as the most prominent target site for nitration with PN. This is evident from the most abundant peak at 24.01 min and related top‐down measurements (Fig. 2A, B_1_). Bottom‐up experiments corroborated this result by the high nitration yield for Tyr 5 represented by a relative peak area of 30.4% for the nitrated peptide relative to all cognate peptides (Fig. 3B_3_). The relative peak area of *^66^nYVK^68^* comprised 31.2% which is even slightly higher (Fig.  3B_3,4_). However, in comparison to other tryptic target peptides that contain Tyr, *^66^YVK^68^* and its nitrated equivalent showed considerably lower signal intensities and tailing (Fig. 3B_1–4_). Both features could thus introduce an uncertainty in the evaluation of the relative abundance. Due to this aspect and based on the top‐down data (Fig. 2A), Tyr 5 is assigned the highest propensity for nitration with PN followed by Tyr 66. Within the top‐down fragment that contained Tyr 120, 150 and 158 (Section 3.2), Tyr 150 showed the highest nitration propensity as revealed by complementary bottom‐up analysis (Section 3.3; Fig. 3B_1_) and is apparently modified in the minor 1x nBet v 1a variant (peak at 24.31 min in Fig. 2A). Most likely, 2x nBet v 1a variants are due to a combinatorial nitration of these high propensity sites. Tyr residues with low nitration propensity, i.e. Tyr 81, 83, 158 (Fig. 3B_1,2_), get obviously modified in higher nitration states. However, their intensities are too low for top‐down analysis.

## 4 Concluding remarks

A laboratory‐built ESI interface was applied in coupling CE to an Orbitrap mass spectrometer. This hyphenation was used for top‐down and bottom‐up characterization as well as analysis of intact nitrated variants of the recombinant major birch pollen allergen Bet v 1a. Nitration occurred at Tyr residues. The maintenance of the CZE method and of the BGE when separating intact variants and tryptic peptides allows for a rapid change between methodic approaches without the need for comprehensive capillary rinsing protocols in between. In top‐down analysis, nitration variants differing only in the localization of the modification were separated. Tyr 5 was the most prominently nitrated residue. A lack of diagnostic fragments in the protein core prevented a differentiation between nitrated Tyr 66, 81 or 83. Equivalently, a lack of fragmentation in the *C*‐terminal protein part impeded a distinction between a nitration at Tyr 120, 150 or 158. Additionally, the averaged nitration propensity of individual Tyr residues was addressed by a bottom‐up approach via the relative intensity of the related nitrated peptide calculated in relation to the summed intensities of the nitrated peptide and its non‐nitrated cognate. Results corroborated and improved the validity of top‐down findings. Tyr 5 was predominantly nitrated followed by Tyr 66 that was nitrated in approximate corresponding relative intensity. Tyr 158 had a smaller nitration tendency. This is plausible due to the surface exposure of Tyr 5 and Tyr 66, whereas Tyr 158 is less accessible. Tyr 81, 83, and 150 showed only a low nitration tendency under the selected reaction conditions. These residues are situated in the interior part of the protein, i.e. a hydrophobic cavity. Tyr 120 takes an even more shielded position and was not found to be nitrated most likely for this reason. HPLC‐ESI‐MS/MS results published recently failed to address both sites that were identified as primary nitration positions with the presented CZE‐ESI‐MS/MS approach after PN treatment: the peptide carrying Tyr 5 was not detected and nitrated Tyr 66 could not be quantified 29. This might be related to the short length or the hydrophilicity of related peptides 22, 29. In addition, different nitration conditions can influence the nitration product 29. The elaborated analytical strategy combines appropriate selectivity in terms of the separation of nitrated intact protein variants and peptides and efficiency of CZE with mass accuracy and mass resolution of LTQ Orbitrap XL MS in orthogonal top‐down and bottom‐up approaches. This provided a deeper understanding of site‐specific nitration of Bet v 1a leading to a comprehensive and improved characterization.

*The authors have declared no conflict of interest*.

## Supporting information

Supporting Information

Supporting Information
