# Supplementary material for: Top‐down and bottom‐up characterization of nitrated birch pollen allergen Bet v 1a with CZE hyphenated to an Orbitrap mass spectrometer
Source: Electrophoresis. 2018 Mar 14;39(9-10):1190–200. doi: 10.1002/elps.201700413 (PMC6175448; doi:10.1002/elps.201700413)
Supplement: Supplementary file 1 — Supporting Information [file ELPS-39-1190-s001.docx]

**Supplement 1: Construction of the SL sprayer**

The coupling device consists of a 0.020 inch thru-hole PEEK cross assembly, three standard fingertight PEEK nuts and one single-winged nut with a 0.24 inch PEEK ferrule (all from IDEX Health & Science, Wertheim, Germany). A bare fused-silica capillary with 450 µm id (670 µm od) from Polymicro Technologies (Phoenix, AZ, USA) was used as an emitter. The protective polyimide layer was thermally removed over a length of 1.5 cm on the outlet side of the emitter capillary. The polyimide layer of the separation capillary was also thermally removed over approximately 1.0 cm at the outlet part. The separation capillary was slightly protruding (0.1 mm) from the emitter. A fused-silica capillary (with 50 µm id and 375 od) for delivering the SL was fixed from the top and a gold wire from the bottom nut of the 0.020 inch thru-hole PEEK cross assembly (see Fig. 1). The gold wire was connected to the high voltage lead of the NSI probe and established the electrical contact. The SL was delivered with 3.0 µL/min by a syringe pump via the PEEK cross assembly.
