# Supplementary material for: Top‐down and bottom‐up characterization of nitrated birch pollen allergen Bet v 1a with CZE hyphenated to an Orbitrap mass spectrometer
Source: Electrophoresis. 2018 Mar 14;39(9-10):1190–200. doi: 10.1002/elps.201700413 (PMC6175448; doi:10.1002/elps.201700413)
Supplement: Supplementary file 2 — Supporting Information [file ELPS-39-1190-s002.docx]

**Supplement 2: Performance evaluation of the ESI sprayer**

Evaluation of the performance of the ESI sprayer was done by analysis of a tryptic digest of nitrated Bet v 1a. Base peak electropherograms (BPEs) recorded with an Orbitrap XL mass spectrometer hyphenated to the CE system via the in-lab designed sprayer for five repetitive injections are shown in Figure S1. Tryptic peptides between 19.5 and 30.0 min were considered since they provided prominent signals. Full scans between m/z 300.00-2000.00 were performed.


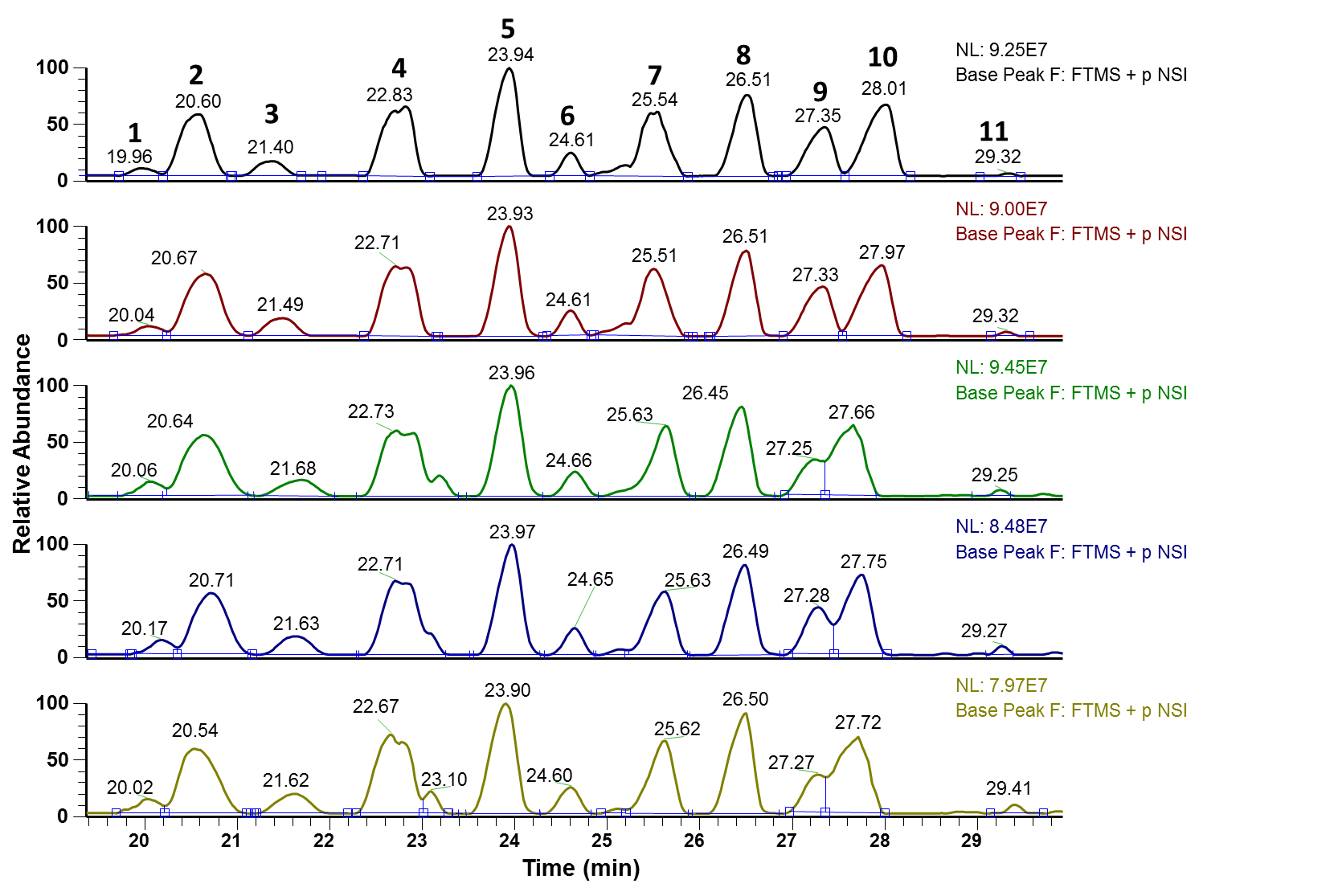


**Figure S1**: Comparison of BPEs representing five consecutive injections of a nitrated Bet v 1a sample digested with trypsin. Further details are given in the experimental section.

Table S2 provides details of the sprayer evaluation. Signal heights and areas of the nitrated peptide *^146^AVESnYLLAHSDAYN^159^* (peak 11 in Figure S1) were not considered (n.c.) due to the low abundance. Peaks 4 and 7 contained two overlapping peptides, respectively. Stated peak areas and heights refer to the sum of both, respectively. As the resolution of peaks 9 and 10 slightly changed over time higher CV for peak height and area are observed (Table S2). The CVs for peak area and height for peak 1 (peptide AEQVK) was higher than for the other peptides, but still <27.0%. This is due to the low signal intensity.

**Table S2: Evaluation of the sprayer performance based on a tryptic digest of nitrated Bet v 1a. Beside the identified sequence of the peptides, their position within the primary protein sequence of Bet v 1a is given. Theoretical (Theor.) and experimental (Exp.) monoisotopic masses are given for [MH^+^]. Identification of nitrated tyrosine (nY) residues and of the deamidation in the variant of peptide 33-54 was done by HCD-MS/MS.**

| **Peak** | **Peptide** | **Position** | **[MH^+^]**  **Exp.** | **[MH^+^]**  **Theor.** | **error**  **[ppm]** | **t_m_**  **[min]** | **t_m_**  **CV [%]** | **Peak area***  **CV [%]** | **Peak height***  **CV [%]** |
| --- | --- | --- | --- | --- | --- | --- | --- | --- | --- |
| 1 | \| AEQVK \| \| --- \| | 130-134 | 574.319 | 574.319 | 0.00 | 20.05 | 0.38 | 25.46 | 26.65 |
| 2 | ISFPEGFPFK | 56-65 | 1168.604 | 1168.602 | -1.71 | 20.63 | 0.32 | 10.82 | 5.20 |
| 3 | ISNEIK | 98-103 | 703.399 | 703.398 | 1.42 | 21.56 | 0.54 | 13.42 | 6.23 |
| 4 | VAPQAISSVENIEGNGGPGTIK  GVFNYETETTSVIPAAR | 33-54  1-17 | 2138.112  1854.924 | 2138.106  1854.920 | 2.81  2.16 | 22.73 | 0.26 | 11.81 | 1.39 |
| 5 | AFILDGDNLFPK | 21-32 | 1349.709 | 1349.708 | 0.74 | 22.94 | 0.11 | 7.06 | 6.80 |
| 6 | GVFN**nY**ETETTSVIPAAR | 1-17 | 1899.910 | 1899.911 | -0.53 | 24.63 | 0.11 | 9.84 | 4.20 |
| 7 | EMGETLLR  VAPQAISSVENIEG**D**GGPGTIK (deamidation) | 138-145  33-54 | 948.482  2139.098 | 948.481  2139.099 | 1.05  -0.47 | 25.59 | 0.22 | 11.47 | 7.29 |
| 8 | AVESYLLAHSDAYN | 146-159 | 1552.728 | 1552.726 | 1.29 | 26.49 | 0.09 | 6.99 | 4.70 |
| 9 | DRVDEVDHTNFK | 69-80 | 1474.688 | 1474.690 | -1.36 | 27.30 | 0.15 | 27.87 | 17.21 |
| 10 | YNYSVIEGGPIGDTLEK | 81-98 | 1854.909 | 1854.909 | 0.00 | 27.82 | 0.57 | 3.86 | 4.21 |
| 11 | AVES**nY**LLAHSDAYN | 146-159 | 1597.719 | 1597.717 | 1.25 | 29.31 | 0.21 | n.c. | n.c. |
